# A randomised study of the impact of the SGLT2 inhibitor dapagliflozin on microvascular and macrovascular circulation

**DOI:** 10.1186/s12933-017-0510-1

**Published:** 2017-02-23

**Authors:** Christian Ott, Agnes Jumar, Kristina Striepe, Stefanie Friedrich, Marina V. Karg, Peter Bramlage, Roland E. Schmieder

**Affiliations:** 1 Department of Nephrology and Hypertension, Friedrich-Alexander-University Erlangen-Nürnberg (FAU), Ulmenweg 18, 91054 Erlangen, Germany; 2 Institute for Pharmacology and Preventive Medicine, Mahlow, Germany

**Keywords:** Arterial remodelling, Pulse wave analysis, Insulin resistance, SGLT2 inhibitors, Blood pressure, Scanning laser Doppler flowmetry, vascular protection

## Abstract

**Background:**

The sodium–glucose cotransporter 2 inhibitor, dapagliflozin, has been shown to improve diabetic control and reduce blood pressure in patients with type 2 diabetes mellitus. Its effects on micro- and macrovascular structure and function have not yet been reported.

**Methods:**

This was a prospective, single-centre, placebo-controlled, double-blind, randomised crossover phase IIIb study conducted between March 2014 and February 2015. After a 4-week run-in/washout phase, patients (N = 59) received 6 weeks of either dapagliflozin 10 mg or placebo once daily. They then underwent a 1-week washout before crossing over to the other treatment. Changes in retinal capillary flow (RCF) and arteriole remodelling were evaluated using scanning laser Doppler flowmetry, while micro- and macrovascular parameters in the systemic circulation were assessed using pulse wave analysis.

**Results:**

Six weeks of dapagliflozin treatment resulted in improvements in diabetes control, including blood glucose and insulin resistance, and reduced office and 24-h ambulatory blood pressure values. RCF decreased from 324 AU at baseline to 308 AU after treatment with dapagliflozin (p = 0.028), while there was little difference after the placebo (318 AU; p = 0.334). Furthermore, the arteriole remodelling that was seen after the placebo phase was not evident after the dapagliflozin phase. Central systolic and diastolic blood pressure values were significantly lower after 6 weeks of dapagliflozin, by 3.0 and 2.2 mmHg, respectively (p = 0.035 and 0.020, respectively vs. baseline).

**Conclusions:**

Six weeks of dapagliflozin treatment resulted in numerous beneficial effects. In addition to achieving superior diabetes control and blood pressure, parameters associated with the early stages of vascular remodelling were also improved.

*Trial registration* http://www.clinicaltrials.gov (NCT02383238)

## Background

The majority of the increased mortality risk associated with type 2 diabetes is a result of cardiovascular disease (CVD). This demonstrates the importance of not only treating hyperglycaemia, but also managing the other contributory risk factors, including hypertension and dyslipidemia [1]. Early changes to the vasculature in patients with type 2 diabetes are characterised by hyperperfusion of microvessels such as those in the eye and kidney, vascular remodelling, and arterial stiffening [2]. Such changes can be identified using non-invasive techniques, even prior to the development of symptoms. The retinal microvasculature can be analysed using scanning laser Doppler flowmetry (SLDF), which provides capillary flow rates and visualisation of vascular structure [3, 4]. Pulse wave analysis, on the other hand, allows evaluation of the compliance of larger vessels through measurement of among others, central pulse pressure (PP), augmentation pressure and index [5–7]. Arterial stiffening and an increased amplitude of wave reflection attributable to peripheral vasoconstriction lead to an earlier return and a higher amplitude of arterial wave reflection in the aorta. This results in an inadequate increase in systolic blood pressure (BP) and a relative decrease in diastolic BP, thus increasing central PP at any given value of mean arterial pressure. Central augmentation index (AIx) is a direct measure of pulse wave reflection. We could previously shown in a non-diabetic cohort across a wide range of BP that both central PP and central AIx correlated well with wall-to-lumen ratio (WLR) of retinal arterioles [8].

Studies evaluating the efficacy of anti-diabetic drugs rarely assess their effect on blood vessels, despite the prognostic significance. Ott et al. demonstrated that the dipeptidyl peptidase (DPP)-4 inhibitor, saxagliptin, was able to reduce retinal capillary flow (RCF) and improve central haemodynamics compared to placebo during a 6-week treatment period [2]. Reductions in arterial stiffness have been demonstrated during treatment with other DPP-4 inhibitors and with pioglitazone added to metformin [9, 10].

Sodium–glucose cotransporter 2 (SGLT2) inhibitors are oral anti-diabetic drugs that work by decreasing re-absorption of glucose in the renal proximal tubule [11]. They have been shown to reduce glycosylated haemoglobin (HbA1c) and fasting plasma glucose (FPG) levels, as well as to induce weight loss and decrease BP [12–14]. This latter effect is likely caused by the simultaneous excretion of sodium, as well as being a consequence of the weight loss. Cherney et al. showed that, in patients with type 1 diabetes, empagliflozin resulted in a reduction in arterial stiffness over the course of an 8-week study [15]. Chilton et al. performed a post hoc analysis of five phase III trials involving patients with type 2 diabetes being treated with empagliflozin [16]. They identified significant reductions in PP and mean arterial pressure as markers of arterial stiffness and resistance, respectively, compared to placebo. Dapagliflozin is another SGLT2 inhibitor; however, while its anti-hyperglycaemic and anti-hypertensive effects have been demonstrated in a number of clinical trials [12] as well as in a real-world care setting [17], there are no available data regarding associated vascular changes.

The present study was performed in order to evaluate the micro- and macrovascular changes that accompany dapagliflozin treatment in patients with type 2 diabetes. Using a combination of non-invasive analytical techniques, we investigated changes in the vasculature after 6 weeks of treatment.

## Research design and methods

### Study design

**Fig. 1:**
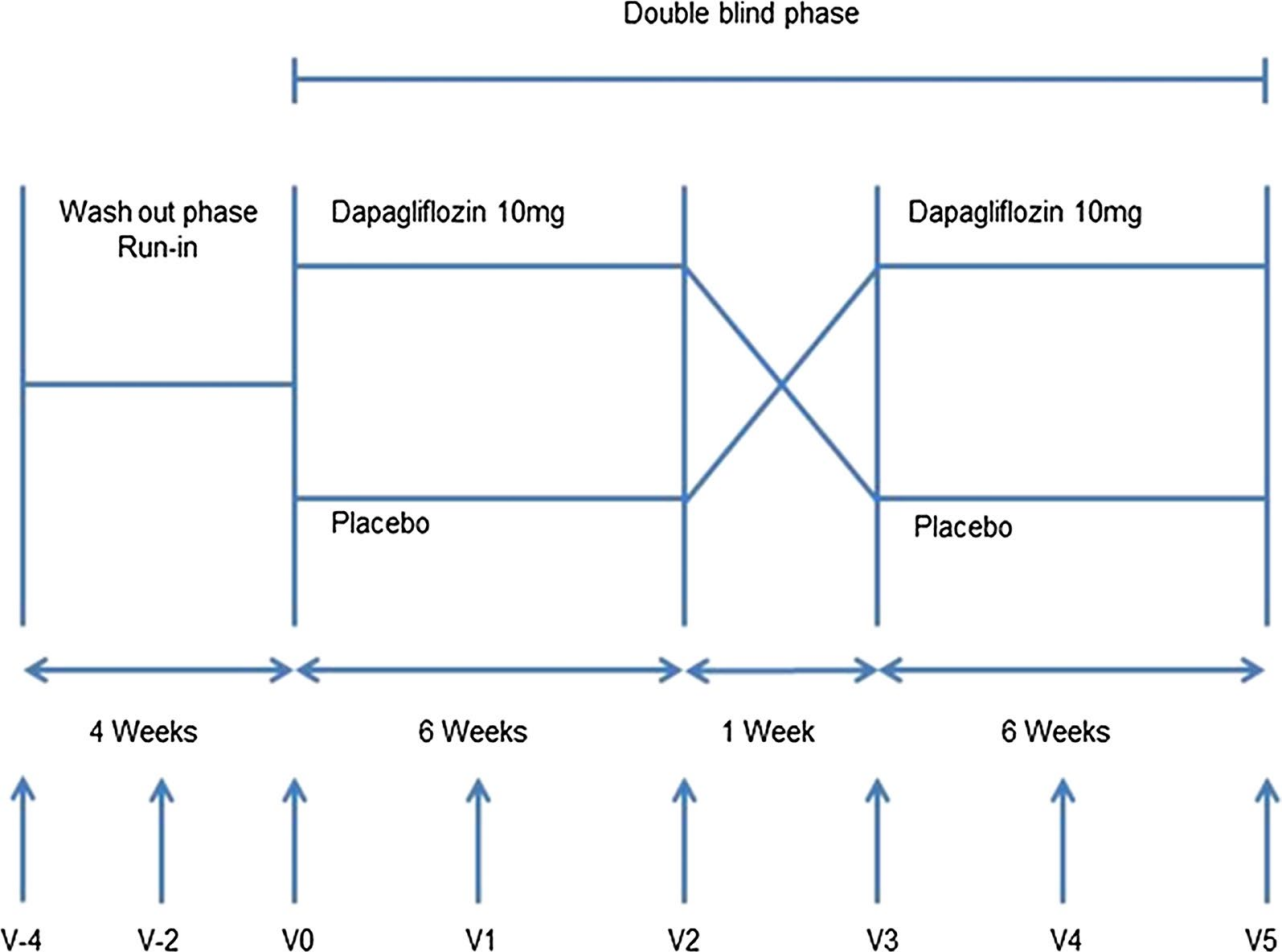
Study design. Micro- and macrovascular parameters assessed at visits 0, 2, and 5. Clinical characteristics assessed at all visits

The study was a prospective, randomised, double-blind, placebo-controlled, cross-over phase IIIb trial performed at the Clinical Research Center of Erlangen-Nuremberg, Germany. Between March 2014 and February 2015, patients with type 2 diabetes were recruited from the University outpatient clinic, through physician referrals, and through the use of newspaper advertisements. After a run-in/washout period (2 weeks for patients not receiving anti-diabetic treatment; 4 weeks for patients treated with anti-diabetic medication), patients were randomised to receive either once-daily oral dapagliflozin 10 mg or placebo through use computer generated algorithm for this single center study (Fig. 1). After 6 weeks, there was a 1-week washout period, and then the patients crossed over to the other treatment.

The study was approved by the Ethics Committee of the University of Erlangen (IRB/IEC) on the 7th February 2014. Furthermore, it was performed in accordance with the Declaration of Helsinki. All patients provided written informed consent prior to inclusion in the study. The study was registered at http://www.clinicaltrials.gov (NCT02383238).

### Study population

Patients were included in the study if they had type 2 diabetes and were between 18 and 70 years of age. Individuals were excluded if they had any other form of diabetes, were being treated with insulin or more than one oral anti-diabetic drug, were being treated with any medication with loop diuretics, had a HbA1c level ≥10% (86 mmol/mol), had a FPG level >240 mg/dl, had BP ≥180/110 mmHg, had an estimated glomerular filtration rate (eGFR) <60 ml/min/1.73 m^2^, or had a body mass index (BMI) >40 kg/m^2^ or suffered from cataract or glaucoma.

### Endpoints

The primary endpoint was the effect of dapagliflozin compared to placebo on RCF and arteriole remodelling after 6 weeks of treatment. Secondary endpoints included the effects on the micro- and macrovascular parameters of the systemic circulation, including central BP, heart rate, and augmentation pressure, as measured by pulse wave analysis. RCF after flicker light exposure was also evaluated serving as a test of vasodilatory capacity of the retinal vascular bed. Other clinical characteristics that were measured were office BP (systolic [SBP] and diastolic [DBP]), 24-h ambulatory SBP and DBP, blood glucose levels, and lipid levels.

### Clinical parameters

Demographic data were recorded at the first visit, prior to the washout period. At the randomisation visit, a fasting blood sample was taken in order to measure HbA1c, FPG, lipid levels, and other biochemical safety parameters (e.g. creatinine, liver enzymes). After consuming a standardised breakfast, further blood was taken to allow measurement of post-prandial glucose (PPG). For evaluation of insulin resistance, the homogenous model assessment (HOMA) index was calculated [18]. Office BP and heart rate measurements were taken in a seated position after 5 min of rest. Twenty-four hour ambulatory BP was measured in parallel with Spacelab 90,207 (Spacelabs Health Care. WA, USA). Measurements were taken every 15 min throughout the day and every 30 min during the night.

SLDF was performed using a Heidelberg retina flowmeter in combination with semiautomatic analysis using Software 4.0 (Welzenbach & Schmieder) [4]. This technique was used to measure microvascular parameters, including RCF, RCF after flicker light exposure (10 Hz; Photo Stimulator 750, Siemens-Elema AB, Sweden), and arteriole dimensions. WLR and wall cross sectional area were calculated in standard manner [2, 4]. Macrovascular parameters were evaluated through pulse wave analysis using the SphygmoCor™ system (AtCor Medical, Sydney, Australia). The obtained central arterial waveforms were used to calculate central PP, augmentation pressure, and AIx.

All biochemical, microvascular, and macrovascular analyses were repeated after each of the two 6-week treatment periods. Any adverse events that occurred during the study were recorded.

### Statistics

The sample size was calculated for the primary endpoint of RCF changes. Using data acquired from a similar study [2], the within standard deviation of RCF was estimated to be 63 AU. In order to show a decrease of 25 AU after dapagliflozin as opposed to placebo after 6 weeks, for α = 0.05 and β = 0.80, the required number of patients was calculated to be 52. From our previous experience, the drop-out rate was estimated to be 15–20% [2]; therefore, we aimed to randomise 62 patients.

Data are presented as absolute values and percentages or means with standard deviation (SD). Statistical significance of differences between the test drug and the placebo was determined using a paired *t* test, assuming normal distribution. Statistical analysis was performed using SPSS release 19.0.

## Results

### Patients

A total of 67 patients were screened, with 62 undergoing randomisation, 31 to initial dapagliflozin and 31 to initial placebo treatment. Of these, 59 patients completed the study and had all the required SLDF and pulse wave analysis data available (full analysis set; FAS). The mean age of the FAS was 60.3 years and 39.0% were female (Table 1). The mean duration of diabetes was 5.54 years and the mean HbA1c level was 6.67% (49 mmol/mol).

**Table 1:** Patient characteristics at baseline

	Mean ± SD or n/N (%)
Age (years)	60.3 ± 7.6
Female	23/59 (39.0)
Weight (kg)	87.4 ± 13
Height (cm)	171 ± 11
BMI (kg/m^2^)	29.9 ± 4.3
Mean duration of diabetes (years)	5.54 ± 4.9
HbA1c	6.67 ± 0.7% (49 mmol/mol)
*Glucose*	
Fasting (mg/dl)	132 ± 28
Postprandial^a^ (mg/dl)	178 ± 66
Fasting Insulin (mU/l)	12.1 ± 7.4
HOMA index	4.02 ± 2.8
*Office blood pressure* (mmHg)	
Systolic	130 ± 14
Diastolic	79 ± 9
*Lipids* (mg/dl)	
LDL-C	143 ± 32
HDL-C	48.2 ± 11
Total cholesterol	207 ± 39
Triglycerides	149 ± 66

N = 59

*BMI* body mass index, *HbA1c* glycosylated haemoglobin, *HOMA* homeostatic model assessment, *LDL*-*C* low-density lipoprotein cholesterol, *HDL*-*C* high-density lipoprotein cholesterol

^a^ A standardised breakfast was given

### Clinical characteristics after 6 weeks

After 6 weeks of treatment, the HbA1c level had not changed significantly from baseline for either the dapagliflozin or placebo, although the final value was slightly lower for the test drug (6.62% [49 mmol/mol] vs. 6.79% [51 mmol/mol]; p < 0.001) (Table 2). In terms of FPG, the level after dapagliflozin treatment decreased by 18 mg/dl (p < 0.001), while that after the placebo did not change from baseline. This resulted in a significantly lower FPG value after the dapagliflozin in comparison to after the placebo (114 vs. 135 mg/dl; p < 0.001). Although both the dapagliflozin and placebo resulted in decreases in PPG, the final values after 6 weeks of treatment were significantly lower for the dapagliflozin (154 vs. 180 mg/dl; p < 0.001). The level of insulin was lower after the dapagliflozin treatment than after the placebo (9.7 vs. 12.9 mU/l; p = 0.002), having decreased by 2.3 mU/l on treatment with dapagliflozin. HOMA index of insulin resistance also changed during each treatment, resulting in a lower value after dapagliflozin and a higher value after placebo. This led to significantly different levels after the 6 weeks (2.77 vs. 4.48 for dapagliflozin and placebo, respectively; p < 0.001).

**Table 2:** Clinical characteristics after 6 weeks of dapagliflozin treatment (mean ± SD)

	Placebo		Dapagliflozin		p value placebo vs. dapagliflozin
	Absolute value (change from baseline)	p value vs. baseline	Absolute value (change from baseline)	p value vs. baseline	
HbA1c (%)^a^	6.79 ± 0.8 (0.12 ± 0.5)	0.064	6.62 ± 0.7 (−0.05 ± 0.3)	0.224	<0.001
*Glucose*					
Fasting (mg/dl)	135 ± 32 (+2.3 ± 18)	0.325	114 ± 19 (−18.3 ± 16)	<0.001	<0.001
Postprandial (mg/dl)^b^	180 ± 67 (+1.2 ± 31)	0.766	154 ± 46 (−24.8 ± 40)	<0.001	<0.001
Insulin (mU/l)	12.9 ± 10.6 (+0.8 ± 5.5)	0.276	9.7 ± 5.4 (−2.4 ± 4.1)	<0.001	0.002
HOMA index	4.48 ± 4.7 (+0.46 ± 2.5)	0.157	2.77 ± 1.7 (−1.25 ± 1.8)	<0.001	0.001
BMI (kg/m^2^)	29.9 ± 4.2 (+0.0 ± 0.7)	0.846	29.5 ± 4.1 (−0.30 ± 0.7)	<0.001	<0.001
*Office blood pressure*					
Systolic (mmHg)	129 ± 13 (−1 ± 10)	0.340	126 ± 12 (−4 ± 12)	0.015	0.102
Diastolic (mmHg)	79 ± 9 (0 ± 6)	0.984	78 ± 9 (−1 ± 6)	0.058	0.113
Heart rate (bpm)	67.8 ± 9.6 (−1.3 ± 6.2)	0.123	68.2 ± 10.6 (−0.8 ± 6.5)	0.332	0.659
*24*-*h ambulatory blood pressure*					
Systolic (mmHg)	129 ± 11 (−1 ± 10)	0.682	126 ± 11 (−3 ± 10)	0.010	0.021
Diastolic (mmHg)	77 ± 7 (0 ± 6)	0.765	75 ± 8 (−2 ± 6)	0.024	0.027
Heart rate (bpm)	75.7 ± 9.5 (+1.4 ± 6.8)	0.172	74.1 ± 7.6 (−0.8 ± 6.0)	0.351	0.132
*Lipids *(mg/dl)					
LDL-C	142 ± 31 (−1 ± 23)	0.688	144 ± 31 (+1 ± 21)	0.808	0.478
HDL-C	48.5 ± 11 (+0.3 ± 4.8)	0.607	48.5 ± 12 (+0.4 ± 4.4)	0.541	0.954
Total cholesterol	207 ± 37 (0 ± 30)	0.937	209 ± 38 (+2 ± 26)	0.584	0.657
Triglycerides	159 ± 100 (+10 ± 91)	0.394	146 ± 74 (−3 ± 63)	0.684	0.043

N = 59

*BMI* body mass index, *HbA1c* glycosylated haemoglobin, *HOMA* homeostatic model assessment, *LDL*-*C* low-density lipoprotein cholesterol, *HDL*-*C* high-density lipoprotein cholesterol

^a^ 6.79% = 51 mmol/mol; 6.62% = 49 mmol/mol

^b^ A standardised breakfast was given

Office SBP decreased by 4 mmHg from baseline after treatment with dapagliflozin, but only 1 mmHg after placebo. DBP was also lower after the drug treatment, but only by 2 mmHg. Twenty-four hour ambulatory BP monitoring also demonstrated reductions in both systolic and diastolic measurements after dapagliflozin treatment, resulting in significantly lower values compared to placebo (SBP: 126 vs. 129 mmHg, p = 0.021; DBP: 75 vs. 77 mmHg, p = 0.027). Lipid levels remained stable during the study, with the only difference being lower triglycerides after dapagliflozin treatment than after placebo (146 vs. 159 mg/dl; p = 0.043).

### Microvascular and macrovascular parameters after 6 weeks of treatment

Retinal capillary flow was lower after 6 weeks of dapagliflozin treatment compared to baseline (308 vs. 318 AU; p = 0.028), while there was no notable change after 6 weeks of placebo (Table 3). RCF after flicker light did not change greatly after either treatment. Mean outer arteriole diameter (AD) remained stable, while there were only non-significant changes in arteriole lumen diameter (LD). However, the WLR ([AD–LD]/LD) after 6 weeks of placebo, an indicator of early vascular remodelling, was slightly higher than at baseline (+0.03; p = 0.034), whereas no such change occurred with dapagliflozin treatment. Arteriolar wall cross sectional area after dapagliflozin treatment did not change (p = 0.800). The same was found for the placebo group (p = 0.210).

**Table 3:** Microvascular and macrovascular parameters after 6 weeks

	Baseline	Placebo		Dapagliflozin		p value placebo vs. dapagliflozin
		Mean ± SD (change from baseline)	p value vs. baseline	Mean ± SD (change from baseline)	p value vs. baseline	
*Microvascular*						
Retinal capillary flow (AU)	324 ± 84	318 ± 87 (−6 ± 45)	0.334	308 ± 78 (−16 ± 53)	0.028	0.157
Retinal capillary flow after flicker, (AU)	351 ± 96	352 ± 101 (+2 ± 65)	0.838	344 ± 115 (−7 ± 84)	0.525	0.477
Outer arteriole diameter (µm)	109 ± 11	109 ± 12 (+1 ± 8)	0.599	109 ± 12 (+1 ± .9)	0.572	0.893
Arteriole lumen diameter (µm)	79.6 ± 7.6	79.0 ± 7.8 (-0.7 ± 6.1)	0.435	80.4 ± 7.8 (+0.7 ± 6.5)	0.410	0.074
Wall/lumen ratio	0.36 ± 0.1	0.39 ± 0.1 (+0.03 ± 0.1)	0.034	0.36 ± 0.1 (+0.01 ± 0.1)	0.524	0.086
Wall thickness (µm)	14.5 ± 3.4	15.1 ± 3.6 (+0.6 ± 3.1)	0.149	14.5 ± 3.3 (0.0 ± 3.1)	0.981	0.096
Wall cross section area (µm^2^)	4338 ± 1291	4531 ± 1373 (+194 ± 1121)	0.210	4378 ± 1331 (+41 ± 1176)	0.800	0.231
*Macrovascular*						
Central systolic BP (mmHg)	121 ± 13	121 ± 13 (0 ± 11)	0.756	118 ± 11 (−3 ± 11)	0.035	0.084
Central diastolic BP (mmHg)	79 ± 8	77 ± 8 (−2 ± 7)	0.050	77 ± 7 (−2 ± 7)	0.020	0.951
Central pulse pressure (mmHg)	42.1 ± 11	43.6 ± 11 (+1.5 ± 8)	0.185	40.9 ± 11 (−1.1± 10)	0.405	0.050
Central heart rate (bpm)	63.9 ± 8.8	63.2 ± 7.5 (−0.7 ± 5.8)	0.398	62.3 ± 7.9 (−1.6 ± 4.5)	0.011	0.359
Augmentation pressure (mmHg)	12.8 ± 5.8	13.3 ± 6.4 (+0.4 ± 3.6)	0.392	12.9 ± 6.0 (+0.1 ± 4.0)	0.918	0.536
Augmentation index	29.3 ± 7.9	29.3 ± 8.3 (0.0 ± 5.1)	1.0	30.1 ± 8.4 (+0.9 ± 5.0)	0.213	0.372
Augmentation index @75^a^	24.0 ± 7.3	23.6 ± 8.1 (−0.4 ± 4.6)	0.556	24.1 ± 8.7 (+0.1 ± 4.6)	0.929	0.603

N = 59

*AU* arbitrary units, *BP* blood pressure

^a^ Normalised to a heart rate of 75 bpm

Slight changes in central SBP and DBP values from baseline led to a lower PP after 6 weeks of dapagliflozin treatment compared to 6 weeks of placebo (40.9 vs. 43.6 mmHg; p = 0.05). Any changes in the placebo phase were not seen. There were no changes in augmentation pressure and index during the study. Furthermore, post-treatment values for central heart rate, augmentation pressure and index did not vary between dapagliflozin and placebo.

## Discussion

Six weeks of treatment with dapagliflozin improved diabetic control and reduced BP in comparison to placebo. Furthermore, the drug was capable of reducing hyperperfusion of retinal capillaries and minimising arteriole remodelling, factors that contribute to the progression of diabetic retinopathy. Pulse wave analysis additionally showed that dapagliflozin reduced markers of arterial stiffness, demonstrating the multiple beneficial effects of the drug.

### Clinical characteristics

There was little change in HbA1c on treatment with either placebo or dapagliflozin, which is likely due to the short period of time (6 weeks) over which the study was carried out. However, the slight increase for placebo and slight decrease for dapagliflozin may indicate an effect of the drug, particularly as FPG and PPG were both significantly lower after the 6 weeks of dapagliflozin treatment. Chronic hyperglycaemia results in reduced insulin sensitivity and reduced β-cell function [19]. In the present study, insulin resistance, as determined by calculating the HOMA index, was significantly lower after 6 weeks of dapagliflozin treatment, while it was slightly higher after 6 weeks of placebo. This finding is in agreement with that reported by Mudaliar et al., where 12 weeks of dapagliflozin treatment resulted in increased insulin sensitivity, while the placebo did not cause a change [20].

Dapagliflozin treatment has previously been shown to reduce BP in patients with type 2 diabetes, likely as a result of its natriuretic and diuretic effects [11]. In a multi-centre study it was shown that the effect on BP was greater in patients who needed a diuretic-like effect to optimize BP control [21]. In the present study, we observed a decrease in office SBP by approximately 4 mmHg after 6 weeks of dapagliflozin treatment, which is of a comparable magnitude to those reported in other studies [11]. As a more accurate measure of BP, we also investigated the effect on ambulatory BP values, and found a significant decrease in both SBP and DBP. Therefore, our data corroborate those previously reported, providing further evidence of the additional benefit of dapagliflozin beyond its glucose-lowering ability.

### Microvascular variables

Microvascular complications of type 2 diabetes are known to be associated with hyperglycaemia, with the UKPDS demonstrating that a 1% decrease in HbA1c resulted in a 37% reduction in the risk of a microvascular complication [22]. There is intensive crosstalk between the mechanisms by which high glucose levels contribute to these problems, with many details remaining unclear. The early stages of diabetic vasculopathy are characterised by vessel hyperperfusion [23]. In patients with poorly controlled type 1 diabetes and background retinopathy, Grunwald et al. found total retinal volumetric blood flow to be 23% higher than that considered normal [24]. In a comparison with patients without diabetes, Patel et al. reported higher blood flow and larger vessel diameter for diabetic patients with signs of retinopathy [25]. Similar changes are observed in the microvascular renal bed, namely renal hyperperfusion and glomerular hyperfiltration. This elevated blood flow is likely to cause vascular damage through increasing shear stress, causing endothelial dysfunction, basement membrane disruption, and extracellular matrix remodelling [23, 26]. Decreasing hyperperfusion would therefore be a significant advantage of any anti-diabetic therapy. In the present study, RCF was found to be significantly lower after 6 weeks of treatment with dapagliflozin, while there was little change after placebo treatment. There could be a number of factors that contributed to this improvement. Firstly, the glucose-lowering effect of the drug could have directly reduced the blood flow. Indeed, Pemp et al. reported increased RCF in patients with type 1 diabetes prior to their morning insulin injection, and that this normalised once glucose levels had been stabilised [27]. In patients with type 2 diabetes, we found that RCF after 6 weeks of treatment with saxagliptin was significantly lower than after 6 weeks of placebo, with this being alongside superior glucose lowering [2]. “In contrast, glucagon-like peptide-1 (GLP-1) therapy, but also sitagliptin, had no effect on capillary perfusion (assessed by nailfold skin capillary videomicroscopy) in patients with type 2 diabetes, suggesting that GLP-1 based therapies in glucose are not mediated through microvascular responses [28]. These data contrast our previous results with saxagliptin [2], other favorable observations with linagliptin [29], and the pathophysiologic rationale that multiple advantageous effects on vascular function structure by GLP-1 based therapies have been demonstrated for patients with type 2 diabetes [30].

As well as affecting RCF, dapagliflozin treatment appeared to prevent changes to the structure of the retinal arterioles. While no significant difference in vessel dimensions were found after the dapagliflozin period compared to baseline, the arteriole wall cross-sectional area and the WLR were both increased after the placebo period. An increased WLR and cross-sectional area are characteristic of the vascular hypertrophy seen in type 2 diabetes [31]. Our findings may, therefore, indicate a positive effect of the dapagliflozin in preventing microvascular remodelling. This is likely to be partly due to the aforementioned reduction in blood flow as a result of the glucose-lowering ability of the drug. Sub-endothelial fibronectin deposition has been shown to be induced by non-laminar flow conditions [32]. This, in turn, would likely alter smooth muscle cell behaviour, potentially resulting in increased proliferation and matrix synthesis [33]. Other glucose-independent effects of the dapagliflozin may also have contributed to preventing arteriole wall thickening. Elevated 24-h SBP has been shown to be independently associated with a higher WLR of retinal arterioles [34]. In the present study, this parameter was lower after 6 weeks of dapagliflozin treatment compared to baseline. Therefore, the reduction in BP may have had a protective effect against wall remodelling.

### Macrovascular variables

Dapagliflozin treatment lowered central SBP, resulting in a reduced PP. On the other hand, while central DBP was lower after treatment with the placebo, there was no change in the central SBP. This resulted in a small but non-significant increase in pulse pressure. These data indicate that the dapagliflozin therapy caused a slight decrease in the stiffness of the aorta and its most proximal branches. The finding of a significant change in stiffness after such a short period of treatment is encouraging and reflects functional changes (i.e. vascular tone, architecture of vascular smooth muscle cell layers) rather than structural ones (i.e. regression of vascular hypertrophy or fibrosis). A number of studies have linked increased central PP to CV morbidity and mortality [35]. This is of particular concern for individuals with type 2 diabetes, who are already at increased risk of CV complications and are more likely to display increased arterial stiffness [36, 37]. Similar to the present study, a number of other publications have reported decreases in arterial stiffness on treatment with anti-diabetic therapy. Duvnjak et al. found that the two DPP-4 inhibitors, sitagliptin and vildagliptin, caused gradual decreases in AIx and central SBP in patients with type 2 diabetes over 12 weeks of treatment [9], while Ott et al. demonstrated lower central SBP after 6 weeks of treatment with saxagliptin [2]. In a cohort of patients with type 1 diabetes, Cherney et al. demonstrated decreases in aortic and carotid AIx from baseline after 8 weeks of empagliflozin treatment [15]. Chilton et al. performed a post hoc analysis of data combined from a number of trials involving patients with type 2 diabetes, and found that empagliflozin significantly reduced PP and mean arterial pressure compared to placebo [16].

In combination with our work on dapagliflozin, the positive results demonstrate that SGLT2 inhibitors are beneficial for decreasing arterial wall stiffness in patients with type 2 diabetes. The mechanism(s) by which these drugs produce these effects are multifaceted. The blood glucose-lowering effect is likely to be one factor. Hyperglycaemia and insulin resistance result in endothelial dysfunction, suppression of nitric oxide synthesis, and production of reactive oxygen species, which are all thought to contribute to arterial stiffening [38, 39]. Schram et al. found lower total systemic arterial compliance not only for patients with type 2 diabetes, but also for subjects with impaired glucose metabolism, in comparison to those with normal glucose metabolism [36]. Rubin et al. found a correlation between hyperglycaemia and markers of arterial stiffness, which held true when considering only the patients without diabetes [40]. The BP-lowering effect of SGLT2 inhibitors is also likely to contribute to the observed decreases in arterial stiffness. Although, a decrease in BP as a result of decreased arterial stiffness cannot be ruled out [41].

## Limitations

The main limitation to this study is its length; however, in the absence of other anti-diabetic therapies, it would not be ethical to administer a placebo for a longer period of time. Further limitations are the small sample size and that findings cannot be extrapolated to type 2 diabetes in general, since patients above 70 years were excluded from the study.

## Conclusions

Six weeks of treatment with the SGLT2 inhibitor, dapagliflozin, resulted in improved diabetic control and reduced ambulatory BP. While these findings have been demonstrated previously, we additionally showed that the drug was capable of reducing hyperperfusion of retinal capillaries and arteriole remodelling, factors that contribute to the progression of diabetic retinopathy. Furthermore, dapagliflozin treatment appeared to reduce arterial stiffness, as indicated by lower central PP. Although the observed effects were small, they demonstrate the multiple advantageous effects on vascular function and structure by dapagliflozin for patients with type 2 diabetes.

## Abbreviations

AD: arteriole diameter
AIx: augmentation index
BMI: body mass index
CVD: cardiovascular disease
DBP: diastolic blood pressure
eGFR: estimated glomerular filtration rate
FAS: full analysis set
FPG: fasting plasma glucose
HOMA: homogenous model assessment
LD: arteriole lumen diameter
PP: pulse pressure
PPG: post-prandial glucose
RCF: retinal capillary flow
SBP: systolic blood pressure
SD: standard deviation
SGLT2: sodium–glucose cotransporter 2
SLDF: scanning laser Doppler flowmetry
WLR: wall-to-lumen ratio
